# Acute Intracerebral Hemorrhage in a Pregnant Woman in the Third Trimester: A Case Report

**DOI:** 10.7759/cureus.92822

**Published:** 2025-09-21

**Authors:** Asma Mustafa Ahmed Ali, Marwa Ahmed Eltayeb Khalid, Ebrahem Mohamed Kher AlYousef, Mayada Yahia Ahmed Elbohouty

**Affiliations:** 1 Obstetrics and Gynecology, Abdullah bin Omran Hospital, Ras al-Khaimah, ARE; 2 Neurological Surgery, Saqr Hospital, Ras al-Khaimah, ARE; 3 Anesthesiology, Abdullah bin Omran Hospital, Ras al-Khaimah, ARE

**Keywords:** american heart association (aha), arteriovenous malformation (avm), cerebral venous sinus thrombosis (cvst), cerebrovascular accident (cva), cincinnati prehospital stroke scale (cpss), computed tomography (ct), hemolysis elevated liver enzymes low platelets (hellp), intracerebral hemorrhage (ich), intracranial pressure (icp), rapid sequence induction (rsi)

## Abstract

Intracerebral hemorrhage (ICH) during pregnancy is a rare but life-threatening condition, with high maternal mortality and long-term neurological risk. We report a case of a 34-year-old normotensive woman at 34+3 weeks’ gestation who presented with sudden neurological decline. Emergency cesarean section was performed due to maternal instability, followed by decompressive craniectomy for a large right-sided ICH. Although no underlying cause was identified, the patient gradually recovered with persistent hemiplegia. This case underscores the need for rapid diagnosis, multidisciplinary intervention, and caution even in the absence of known risk factors.

## Introduction

Pregnancy-related stroke affects approximately 30 per 100,000 pregnancies, with nonhemorrhagic subtypes such as arterial ischemic stroke and cerebral venous sinus thrombosis (CVST) accounting for 19.9 cases and hemorrhagic strokes accounting for 12.2 cases per 100,000 pregnancies [1]. Hypertensive disorders of pregnancy including preeclampsia, eclampsia, and HELLP (Hemolysis, Elevated Liver enzymes, Low Platelet count) syndrome are among the most significant contributors to intracerebral hemorrhage (ICH), markedly increasing cerebrovascular risk during both antepartum and postpartum periods [2]. The long-term risk of ICH is elevated approximately 3.7-fold in women with gestational hypertension and 8.2-fold in those with preeclampsia [3].

While the early postpartum period has traditionally been considered the peak risk window, emerging evidence identifies the third trimester as a particularly vulnerable phase, especially in women of advanced maternal age or those with abnormal placentation [4]. Notably, 20-30% of pregnancy-associated ICH cases occur in the absence of identifiable vascular malformations or chronic hypertension, underscoring the distinct hemodynamic and coagulative shifts that characterize pregnancy [1,5].

This case illustrates the diagnostic and therapeutic challenges of managing ICH in late pregnancy, highlighting the importance of prompt clinical recognition, timely neuroimaging, and coordinated multidisciplinary intervention to improve maternal and fetal outcomes.

## Case presentation

A 34-year-old Indian woman, gravida 4 para 3, with three previous normal vaginal deliveries and one neonatal death due to renal anomalies, presented with no history of hypertension. She reported contracting chickenpox at seven months of gestation. She was brought to the emergency department by ambulance following a sudden episode of loss of consciousness at home. During transport, she regained partial consciousness but remained confused. The ambulance team noted hypotension, left facial droop, left hemiparesis, and slurred speech. Upon arrival at the hospital, she was conscious but appeared dizzy and weak. Initial vital signs were as follows: temperature 36.9°C, heart rate 76 bpm (regular), respiratory rate 18 breaths/min, blood pressure 97/54 mmHg, oxygen saturation 100% on room air, and Glasgow Coma Scale (GCS) score of 14/15. Neurological examination revealed inability to raise or move the left arm, no grip strength in the left hand, and lateral deviation of the right eye pole; she met the criteria of CPPSS (Cincinnati Prehospital Stroke Scale).

Abdominal examination revealed a gravid uterus at 36 weeks' gestation with no uterine contractions. Speculum examination showed a closed cervix and no vaginal bleeding. Bedside obstetric ultrasound demonstrated a viable fetus in cephalic presentation, normal amniotic fluid volume, anterior upper placenta, and no signs of placental abruption. Gestational age was estimated at 34 weeks and 3 days.

Ten minutes after arrival, a neurological consultation was arranged, and while preparing the patient for brain radiological imaging, the patient’s condition deteriorated rapidly. She developed hypoxia (SpO₂ dropped to 85%), and oxygen therapy was initiated via a simple face mask. Neurologically, her GCS score fell to 5/15, accompanied by anisocoria: the right pupil measured 6 mm and was non-reactive, while the left pupil measured 3 mm and was sluggishly reactive. She exhibited new-onset left-sided hemiplegia, left facial droop, aphasia, frothy oral secretions, and jerky movements of the lower limbs. She also complained of a sudden, severe headache and became increasingly irritable. Repeated vital signs showed hypotension (BP 89/53 mmHg), mild tachycardia (HR 92 bpm), and improved oxygen saturation following oxygen supplementation.

**Figure 1:**
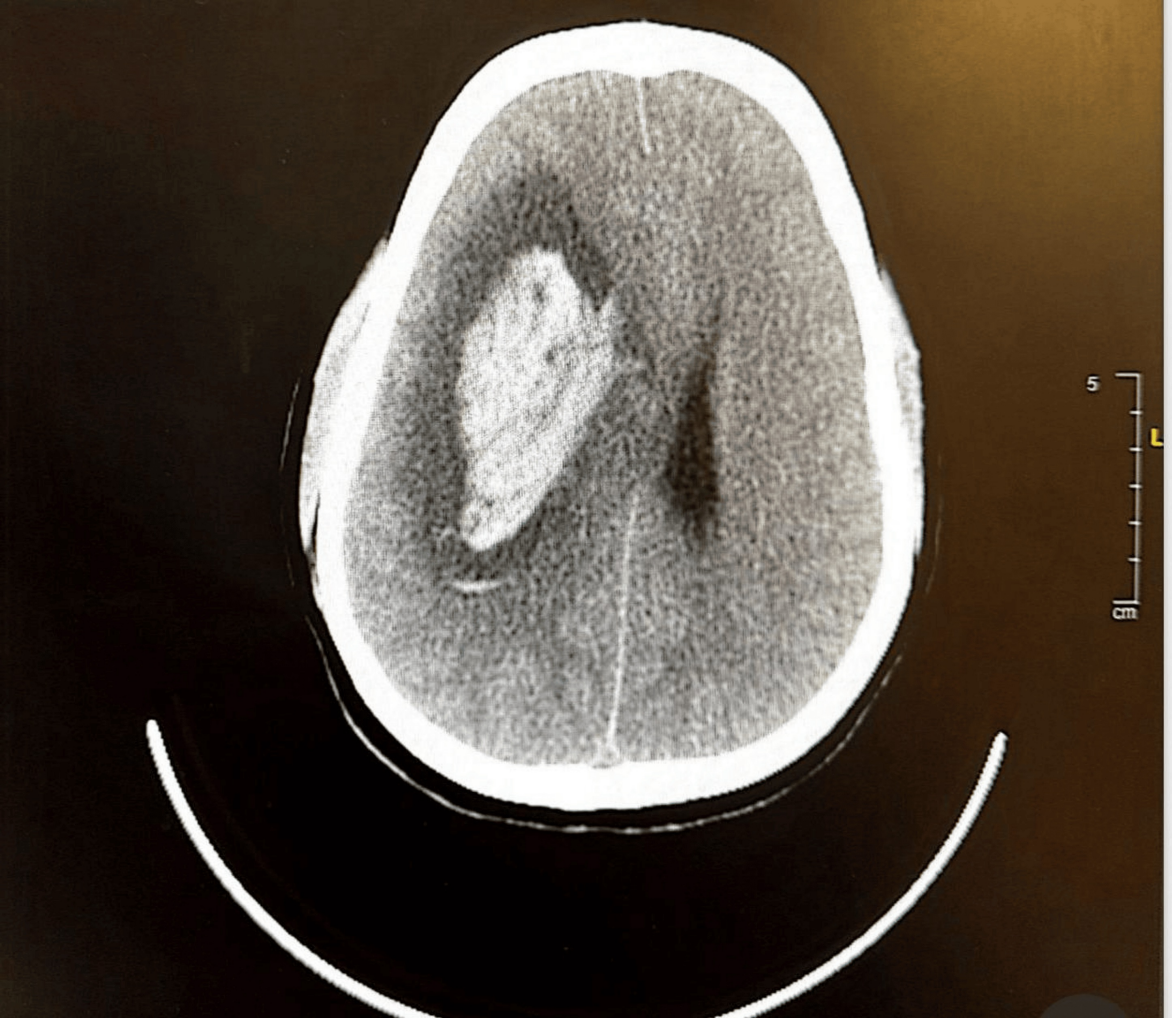
CT of the brain at the time of presentation

Following evaluation by a multidisciplinary team comprising an anesthetist and obstetrician, and in view of the patient’s rapid neurological decline and risk of cardiopulmonary arrest, the decision to perform an emergency category 1 lower segment cesarean section was deemed necessary and was promptly undertaken within 2 hours of the patient’s arrival. Intraoperative anesthetic care was guided by a highly interactive, real-time multidisciplinary approach involving continuous coordination between the anesthesiologist and obstetrician. The main goals were to optimize maternal cerebral perfusion, prevent secondary brain injury, and ensure safe fetal delivery. General anesthesia was induced via rapid sequence induction (RSI) using titrated propofol (for hemodynamic stability) and rocuronium (to provide rapid neuromuscular blockade), minimizing the risk of aspiration and avoiding increases in intracranial pressure (ICP). Preoxygenation was carefully performed to maintain maternal oxygen reserves and avoid hypoxemia. Delivery occurred within minutes after incision, minimizing fetal exposure to anesthetic agents. A live male infant weighing 2,160 grams was delivered, with Apgar scores of 1 at 1 minute and 5 at 10 minutes. The neonate was transferred to the NICU for close observation and monitoring and was discharged later in good health. Following delivery, uterotonic agents were administered to ensure uterine contraction, with caution to avoid sudden hemodynamic shifts. Intraoperatively, a retroplacental hematoma (100 g) was discovered. Hemodynamic parameters were meticulously maintained within target ranges using invasive arterial blood pressure monitoring. Capnography was used to maintain normocapnia (PaCO₂ 35-40 mmHg), preventing both cerebral vasodilation and vasoconstriction. Anesthetic maintenance was achieved with a balanced technique using low-dose volatile agents and short-acting opioids (e.g., fentanyl), avoiding excessive cerebral vasodilation while ensuring adequate anesthesia depth, with bispectral index (BIS) kept in the range of 40 to 60. Throughout the procedure, the anesthesia team maintained continuous communication with obstetricians and neurosurgeons to anticipate post-cesarean needs. Postoperatively, the patient was transferred intubated and sedated with intravenous continuous infusion of alpha 2 agonist (dexmedetomidine), with a portable ventilator and standard monitoring, to the radiology department for urgent computed tomography (CT) of the brain, which revealed a large acute intracerebral hematoma in the right deep parietal periventricular region measuring 3.9 × 7 × 5.7 cm in its maximum axial and craniocaudal dimensions (Figure 1).

**Figure 2:**
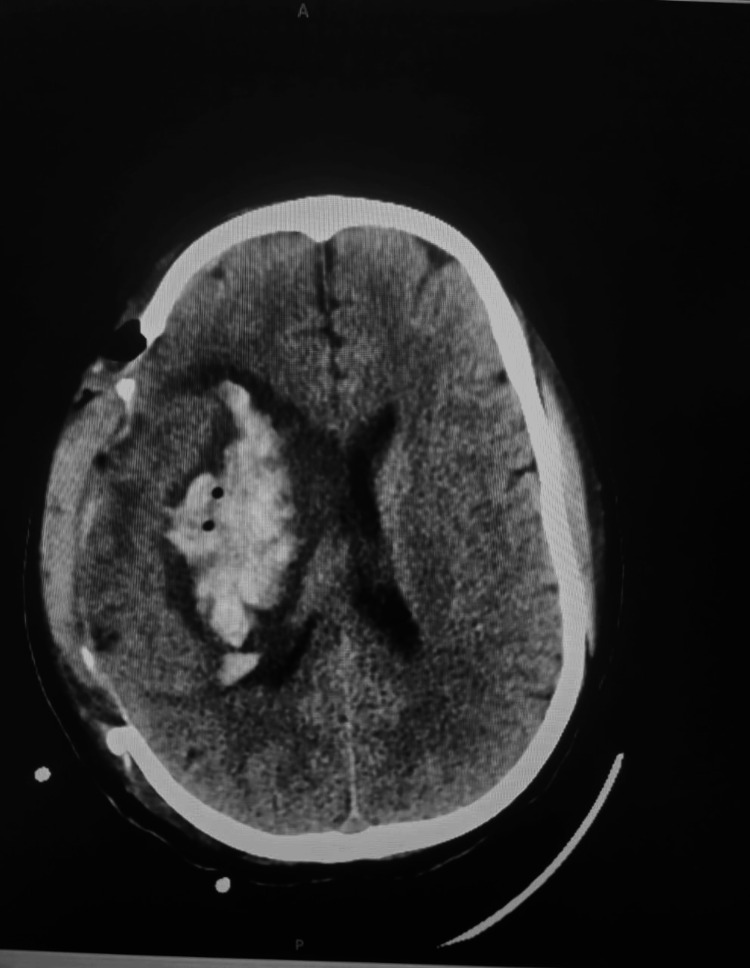
Follow-up CT of the brain on day 3 post-craniectomy

**Figure 3:**
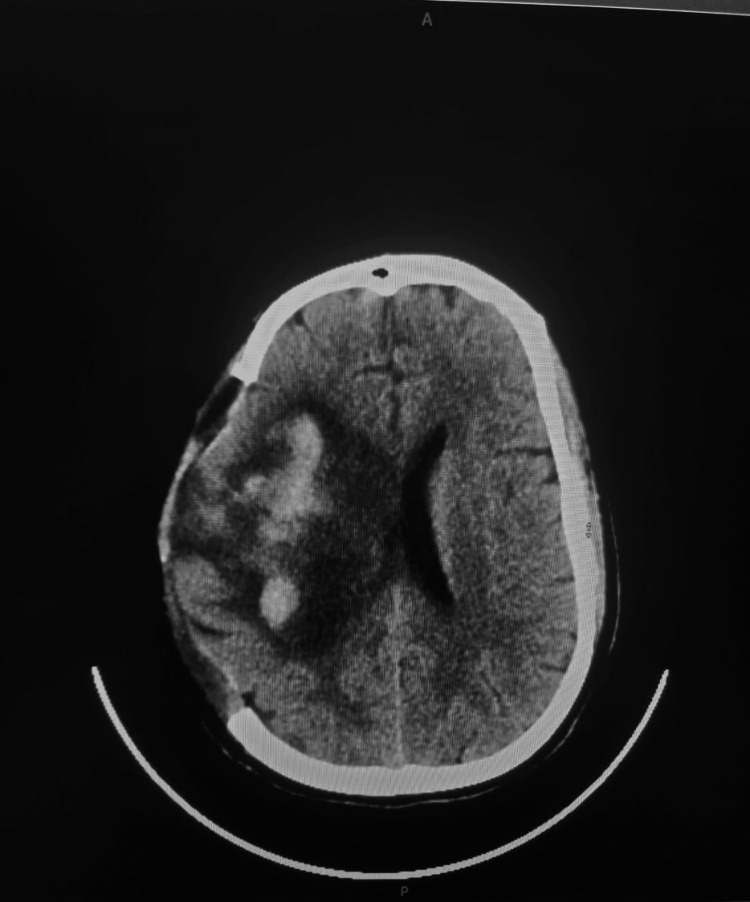
Follow-up CT of the brain on day 8 post-craniectomy

The patient was then transferred to the neurosurgical department, where a decompressive craniectomy was urgently performed to manage raised ICP. Intraoperatively, a large clot (150 g) was removed piecemeal, after which brain pulsations resumed. A ventriculoperitoneal shunt was placed to relieve elevated ICP. Postoperatively, the patient was admitted to the intensive care unit in a hemodynamically stable condition. The patient’s neurological status gradually improved, allowing for successful extubation. However, she experienced both urinary and fecal incontinence. Follow-up CT of the brain (Figures 2, 3) showed regression of the midline shift of brain structures to the left. She was enrolled in a structured physiotherapy program to support ongoing neurorehabilitation.

After two weeks, she was discharged with stable vital signs, a GCS score of 11/15, and persistent left-sided hemiplegia. She tolerated nasogastric tube feeding without difficulty and remained with an indwelling urinary catheter. She was discharged on levetiracetam 500 mg twice daily for six months. Unfortunately, follow-up was lost as the patient returned to her home country.

After one and a half years in her home country, we received feedback from the patient’s family indicating that she continued to experience urinary and fecal incontinence four months after surgery. However, she is now continent with both urine and feces. She remained with persistent left-sided hemiplegia and was under neurological and physiotherapy care.

## Discussion

ICH during pregnancy, although infrequent, constitutes a critical neurological emergency with profound consequences for both mother and fetus. Traditionally linked to hypertensive disorders such as preeclampsia, eclampsia, and HELLP syndrome, ICH may also arise in the absence of overt clinical risk factors, complicating timely recognition and management. This underscores the importance of maintaining a high index of suspicion and adopting a multidisciplinary approach to maternal neurological deterioration, regardless of baseline blood pressure status.

In our case, a 34-year-old gravida 4 woman with no prior history of hypertension presented in the third trimester with acute neurological deterioration. Her case aligns with emerging data suggesting that the third trimester carries comparable cerebrovascular risk to the early postpartum period, particularly in women with abnormal placentation or advancing maternal age [4,6]. The intraoperative finding of a retroplacental hematoma raises suspicion for a concealed hypertensive disorder, such as placental abruption or HELLP syndrome, despite the absence of overt clinical signs.

While our patient was just below the conventional threshold for advanced maternal age, recent evidence suggests that cerebrovascular risk begins to rise as early as age 30, particularly when compounded by placental pathology or hypertensive disorders [3,6]. This highlights the need for individualized risk stratification that goes beyond age alone.

Importantly, 20-30% of pregnancy-related ICH cases occur without identifiable vascular malformations or chronic hypertension [1,2,5]. In our patient, neuroimaging excluded aneurysm and arteriovenous malformation (AVM), consistent with findings that pregnancy does not significantly increase AVM rupture risk, although AVMs are implicated in up to 20% of cases [4]. This supports the hypothesis that pregnancy-specific physiological changes including increased blood volume, hormonal fluctuations, and hypercoagulability may independently precipitate hemorrhage in predisposed individuals.

The patient’s presentation - sudden loss of consciousness, hemiparesis, and rapid decline in GCS score - is characteristic of large volume lobar or deep hemorrhage. Non-contrast CT remains the gold standard for rapid differentiation between ischemic and hemorrhagic stroke in pregnancy, and maternal stabilization must take precedence over concerns about fetal radiation exposure [7,8].

Management of ICH in pregnancy demands rapid, coordinated multidisciplinary action. In this case, simultaneous involvement of obstetrics, anesthesia, and neurosurgery enabled a timely category 1 cesarean section followed by decompressive craniectomy. This approach reflects best practice recommendations for maternal neurological deterioration in late pregnancy, where urgent delivery may improve both cerebral perfusion and neonatal outcomes [7,9].

Anesthetic management in such scenarios is uniquely challenging. General anesthesia with RSI is preferred to minimize aspiration risk and ensure airway control in neurologically unstable patients [7]. Key goals include maintaining cerebral perfusion, avoiding fluctuations in ICP, and ensuring normocapnia and adequate oxygenation [7,8]. In our case, titrated propofol and rocuronium were used to achieve hemodynamic stability, with intraoperative monitoring guided by invasive arterial pressure, capnography, and BIS. The anesthetic strategy was tailored to balance neuroprotection with safe fetal delivery, and continuous communication between teams ensured readiness for immediate neurosurgical intervention.

Despite timely intervention, maternal morbidity remains high. Long-term studies report that 60-75% of survivors of pregnancy-associated ICH experience persistent neurological deficits [2]. Our patient was discharged with left-sided hemiplegia and transient incontinence, though gradual recovery of continence was later achieved. This outcome reflects the enduring impact of ICH, even with optimal care.

## Conclusions

Pregnancy-associated ICH, though rare, represents a critical obstetric emergency with high potential for maternal and fetal morbidity and mortality. This case underscores the importance of maintaining a high index of suspicion for ICH in pregnant patients presenting with acute neurological symptoms, even in the absence of traditional risk factors such as chronic hypertension or known vascular anomalies. Hypertensive disorders of pregnancy, including preeclampsia and HELLP syndrome, remain the most common and preventable contributors to ICH. Timely neuroimaging, multidisciplinary coordination, and individualized risk assessment are essential for optimizing outcomes. Surgical interventions such as decompressive craniectomy, when appropriately timed, may offer life-saving benefits in cases of elevated ICP. Persistent neurological deficits, as seen in this patient, reflect the need for long-term rehabilitation and follow-up. Future strategies should focus on early recognition of hypertensive complications, closer monitoring of high-risk pregnancies, and continued education of obstetric and emergency teams on the atypical presentations of cerebrovascular events in pregnancy.
